# Procedural and patient-related factors play differential roles in postdural puncture headache

**DOI:** 10.55730/1300-0144.5987

**Published:** 2025-01-26

**Authors:** Ece YANIK, Doğa VURALLI, Tuğba TUNÇ

**Affiliations:** 1 Department of Neurology and Algology, Faculty of Medicine, Gazi University, Ankara, Turkiye; 2 Neuropsychiatry Center, Gazi University, Ankara, Turkiye; 3 Neuroscience and Neurotechnology Center of Excellence (NÖROM), Gazi University, Ankara, Turkiye

**Keywords:** Postdural puncture headache, headache, osmophobia, idiopathic intracranial hypertension, onset time of postdural puncture headache

## Abstract

**Background/aim:**

The incidence of postdural puncture headache varies depending on the multifactorial nature of the risk factors. Patient-related and procedure-related risk factors are still controversial. In this prospective observational study, we aimed to evaluate the risk factors for postdural puncture headache, the effect of previous headache features on the development of postdural puncture headache, and the risk factors for the immediate or delayed postdural puncture headache.

**Materials and methods:**

We prospectively analyzed 116 patients who underwent lumbar puncture for diagnostic or therapeutic purposes. All clinical and laboratory findings, previous headache history, and the features and information related to the lumbar puncture procedure were evaluated.

**Results:**

We found that the presence of longer and more frequent attacks, independent of the type of the prior headache, and a history of migraine accompanied by osmophobia are independent risk factors for postdural puncture headache. A lower volume of cerebrospinal fluid collected, higher frequency of previous headache attacks, diagnosis of idiopathic intracranial hypertension, and acetazolamide use were risk factors for the immediate-onset postdural puncture headache.

**Conclusion:**

This is the first study to detail the previous headache features in the development of postdural puncture headache and to extensively examine the risk factors for immediate and delayed postdural puncture headache. It was shown for the first time that patient-related factors may be more important in terms of the development of postdural puncture headache, and procedure-related factors gain importance in terms of its time of occurrence.

## 1. Introduction

Postdural puncture headache (PDPH) [1] is the most common complication associated with lumbar puncture (LP) [1]. The definition of PDPH according to the International Classification of Headache Disorders (ICHD-3) is ‘Orthostatic headache occurring within five days of a lumbar puncture, caused by cerebrospinal fluid (CSF) leakage through the dural puncture. It is an orthostatic headache usually associated with neck pain, tinnitus, hearing loss, photophobia and/or nausea. It remits spontaneously within two weeks, or after sealing of the leak with autologous epidural lumbar blood patch’ [2]. The mechanism of PDPH is uncertain, but various theories have been proposed [3]. The downward stretch of pain-sensitive structures secondary to CSF volume loss is the most common proposed theory. Other theories include intracranial vasodilation compensating for CSF loss (Monro–Kellie–Burrows doctrine), hypersensitivity to substance P, upregulation of NK1 receptors, and intracranial hypotension due to CSF volume depletion and/or ongoing CSF leak from dural and arachnoid injury [4–6]. Additionally, differential compliance within the caudal and rostral segments of the CSF system may contribute to intracranial hypotension [7].

PDPH incidence ranges from 4% to 50%, influenced by a spectrum of multifactorial risk factors [8]. Independent risk factors have been identified as female sex, age between 31 and 50 years, a prior history of PDPH, and the orientation of the needle bevel perpendicular to the spinal column’s longitudinal axis [4]. The correlation between other patient-related and procedure-related risk factors—such as body mass index, history of previous headache, volume of CSF collected, number of needle insertions, procedural traumatization, and CSF opening pressure—exhibits variability across studies [1]. Limited retrospective studies on the risk factors for PDPH onset have suggested that different risk factors may be present for immediate and delayed PDPH [1,9].

In the present prospective observational study, we aimed to evaluate the risk factors for PDPH, the features of previous headaches, and their effect on the development of PDPH, as well as the risk factors for the development of PDPH within the first 24 h (immediate) or after 24 h (delayed).

## 2. Materials and methods

All patients subjected to LP for diagnostic or therapeutic purposes were recruited from Gazi University Faculty of Medicine, Neurology inpatient clinic between January and June 2021. The inclusion criteria for patients who underwent LP were as follows: 1) being between 18 and 75 years of age and 2) being conscious and able to answer the questions for the prospective analysis. Only the first LP procedure among the repeated LPs in the same patient was evaluated. Patients were excluded if 1) they had decreased level of consciousness within 1 week after the LP procedure and were not able to describe whether or not they had PDPH, 2) they could not be contacted during follow-up, or 3) they had missing procedural data.

The clinical and laboratory (serum and CSF) findings of the patients and procedural data of the LP were recorded. The prior headache details of the patients were evaluated by two headache specialists (TT, DV). All previous headache features (such as the number of monthly headache days, duration and location of the headache, accompanying symptoms, intensity and duration of the headache attacks, headache days with analgesic intake), anxiety about the procedure ( 5-point Likert scale, from 1 = not at all to 5 = extremely), and LP indications were recorded. The patients were evaluated for PDPH within the first 7 days after LP (bedside or telephone visits).

A single type of needle (Quincke 18 G, 90 mm) was used due to its widespread use in our clinic. The patients were evaluated after the LP was performed. Procedural data were collected and recorded after the procedure. After the LP procedure, PDPH was diagnosed according to the International Classification of Headache Disorders (ICHD-3) diagnostic criteria for primary headaches (migraine, tension-type) and idiopathic intracranial hypertension (IIH) [2]. In cases in which PDPH was identified, all attributes (including severity, duration, onset time, accompanying symptoms, complications, and treatment) were recorded.

Further analysis was performed to divide the patients with PDPH into two groups according to the timing of symptom onset: the first 24 h and after 24 h. These groups were then reevaluated in terms of all identified risk factors.

## 3. Statistical analysis

The data were analyzed using IBM SPSS Statistics 17.0 (IBM Corporation, Armonk, NY, USA). The distribution of continuous numerical variables was examined for normality using the Kolmogorov–Smirnov test. Descriptive statistics were expressed as mean ± standard deviation or median (25th percentile–75th percentile) for continuous numerical variables, while categorical variables were presented as percentages. The significance of differences between the two groups was evaluated using Student’s t-test when the data were normally distributed, whereas the significance of difference in continuous numerical variables between the two groups was assessed using the Mann–Whitney U test when the data were non-normally distributed. Unless otherwise specified, Pearson’s chi-squared test was used for the analysis of categorical data. However, in 2 × 2 contingency tables, if at least one-fourth of the cells had an expected frequency below 5, the respective categorical data were evaluated using Fisher’s exact test; if the expected frequency was between 5 and 25, the continuity corrected chi-squared test was applied. Moreover, for R × C contingency tables (where at least one of the categorical variables in the rows or columns had more than two outcomes), if at least one-fourth of the cells had an expected frequency below 5, the respective categorical data were assessed using the Fisher–Freeman–Halton test. The potential factors that could distinguish between the groups with and without PDPH, as well as within the PDPH group to differentiate patients according to the onset times of PDPH, were investigated using multivariate logistic regression analyses. Variables identified with a p-value <0.10 in the univariate statistical analyses were included as candidate factors in the regression models. Subsequently, the final model containing the most determinant variables for distinguishing the groups was achieved using backward stepwise elimination (backward LR). Odds ratios and 95% confidence intervals were calculated for each variable. The results were considered statistically significant at p < 0.05.

## 4. Results

A flowchart of the study is given in the Figure. Our study included 116 participants between the ages of 19 and 74, with an equal distribution of males and females. The demographic characteristics are detailed in Table 1 and comorbidities and medications are detailed in Table 2. A total of 69 participants (59.5%) reported experiencing headaches before the procedure. Based on the ICHD-3 diagnostic criteria, the distribution of headache types among these individuals was as follows: 44.9% migraine, 17.4% tension-type headache, 13% IIH, 11.6% other headache disorders, and 7.2% co-occurrence of migraine and IIH. The characteristics of the previous headaches are presented in Table 3.

Post-procedure, none of the patients encountered serious complications. Headaches were reported by 66 patients (56.9%) following the procedure. When headache characteristics were evaluated as PDPH according to ICHD-3 criteria [2], 44.8% of all LP patients and 80% of patients with headache complaints had PDPH.

As shown in Table 4, as a result of multivariate backward stepwise elimination logistic regression analysis, the most important determining risk factors for PDPH, respectively were younger age, female sex, having previous migraine accompanied by osmophobia, and a longer duration of previous headache attacks. Additionally, frequent headache attacks (p = 0.04), high anxiety score (p = 0.03), few LP attempts (p = 0.01), high CSF chloride level (p = 0.01), low ferritin level (p = 0.01), low serum triglyceride level (p = 0.03), and topiramate use (p = 0.04) were found to be risk factors for the development of PDPH.

Independent of other factors, having a history of migraine accompanied by osmophobia increased the risk of developing PDPH statistically significantly by 3.4-times (95% confidence interval (CI): 1.0–11.5) (p = 0.05). In addition, 64% of the patients who also had osmophobia had a high frequency of migraine attacks.

The probability of developing PDPH continued to increase as the duration of headache attacks increased (odds ratio = 1.609; 95% CI: 1.024–2.530 and p = 0.04). Compared to all other possible factors, each 10-year decrease in age significantly increased the likelihood of developing PDPH and was the most definitive risk factor (odds ratio = 1.451; 95% CI: 1.1–2.0 and p = 0.02). Independent of other factors, the probability of PDPH occurring in women was significantly 2.5 times higher than in men (95% CI: 1.1–5.7 and p = 0.03) (Table 3). The main determinants of PDPH, such as sex and age, were not found to have an effect on immediate-onset PDPH.

The association between topiramate use (p = 0.04) and PDPH was found to be statistically significant, but for other drugs, including acetazolamide (p = 0.24), there was no significant association. The risk factors differed between immediate- and delayed-onset PDPH. A lower volume of CSF collected (every 1 cc), pre-LP diagnosis of IIH compared to those without pre-LP headache, and use of acetazolamide were specific risk factors for immediate-onset PDPH. The development of immediate-onset PDPH continued to increase significantly with each 1 cc less CSF drained (odds ratio = 1.155; 95% CI: 1.0–1.3 and p = 0.03).

There was no significant correlation between the previous headache type and the development of PDPH (p = 0.15). Moreover, there were no significant correlations between preliminary diagnosis of LP, comorbidities, and the development of PDPH (Table 1). Compared to the patients who did not have headaches before LP, having a pre-LP diagnosis of IIH significantly increased the likelihood of immediate-onset PDPH by 10.2 times. No such difference was observed for other headaches.

The mean anxiety score was significantly higher in patients with PDPH (3.7 ± 1.0) than in those without PDPH (3.2 ± 1.0) (p = 0.03).

High CSF chloride (p = 0.01) levels and low serum ferritin (p = 0.01) and triglyceride (p = 0.03) levels were also risk factors for the development of PDPH. No significant correlation was found between other CSF and serum parameters and the development of PDPH.

Except for the number of LP attempts, none of the other procedure characteristics significantly affected the development of PDPH. The number of attempts per LP session was inversely correlated with the risk of PDPH, and the risk of PDPH decreased as the number of attempts increased (p = 0.01). The collected CSF volume was a minimum of 5 cc and a maximum of 30 cc with a median of 14.5 cc (95% CI: 10–18.7). CSF opening pressure was measured in 44 patients (37.9%) and was normal in 79.5% and high in 20.5%. CSF pressure had no statistically significant effect on the development of PDPH (p = 0.71). According to the records, 97.4% of the patients were in lateral decubitus position and 2.6% were in sitting position during LP. A previous LP procedure increased the risk of developing PDPH compared to the first time LP procedure, but it did not reach statistical significance (p = 0.1). Procedure-related features are shown in Table 5.

## 5. Discussion

In the present study, we evaluated the risk factors for PDPH, the effect of all previous headache features on the development of PDPH, and the risk factors for the development of immediate and delayed PDPH in detail. Our study was the first to examine the effects of all previous headache features on PDPH. Even though earlier studies showed that the history of pre-existing headaches increases the risk of PDPH up to 3 times [6,10,11], they did not report a clear association between the previous headache type and headache characteristics and the development of PDPH. In our study, the presence of a history of migraine accompanied by osmophobia was identified as an independent risk factor for the development of PDPH. Additionally, we found that the risk of developing PDPH increased with longer prior headache attack duration and higher monthly prior headache frequency, regardless of the preexisting headache type. High CSF chloride level, topiramate use, low serum ferritin, and triglyceride levels were also risk factors for PDPH. The main risk factors for the development of acute onset PDPH were a higher number of prior headache attacks per month, a lower volume of CSF collected, a diagnosis of IIH, and a history of acetazolamide use. Our study indicated that patient-related factors could be more critical in terms of PDPH development, and procedure-related factors could be more important in terms of PDPH onset time.

A significant association between osmophobia and PDPH was shown for the first time in our study. Osmophobia was observed only in migraine patients and was observed in 20.3% of all patients who developed PDPH and in 38.8% of migraine patients. Osmophobia is an additional symptom that shows high specificity in distinguishing migraine from other primary headaches [12,13]. In our study, this significant increase in patients with migraine-specific symptoms such as osmophobia may be supportive of central sensitization, which is also discussed in the pathophysiology of PDPH. Additionally, in the literature, osmophobia is found to be more common among chronic migraine patients compared to in episodic migraine patients [14]. In support, the average attack frequency is higher in osmophobic patients and osmophobia is observed more frequently in allodynic migraineurs [14]. We found that the risk of developing PDPH increased as the attack duration and the frequency of the previous headaches increased, regardless of the headache type. As the headache attack duration increased, the probability of developing PDPH increased significantly.

This is the first study in which PDPH developing within the first 24 h (immediate) and after 24 h (delayed) was evaluated in terms of all risk factors. It was observed that a lower volume of CSF collected (for each 1 cc) significantly increased the probability of developing immediate onset PDPH. Having a pre-procedural headache with IIH features increased the likelihood of immediate-onset PDPH compared to having no headache history. No similar effect was observed in other headache types. The high frequency of previous headache attacks and the use of acetazolamide significantly increased the risk of developing immediate-onset PDPH.

In the present study, PDPH developed during the first 24 h in all patients who took acetazolamide, and all of these patients had IIH. Our series displayed significantly greater immediate-onset PDPH intensity in the IIH patient group (10 times higher than in those without headaches). Chronic long-term high intracranial pressure can impair central nervous system (CNS) adaptation. The intracranial meninges may become sensitized due to the mechanical stimuli of repetitive abnormal CSF pressure pulsations in IIH [15,16]. Therefore, IIH may alter CNS compliance after a while, causing an increase in the CSF pressure gradient between the caudal and cranial compartments. This may mean that even a moderate pressure variation will be less tolerated after LP [7]. It is essential to consider these results when there is a headache after the LP procedure to prevent misdiagnosis and incorrect treatment protocols as exacerbation of the IIH symptoms. Studies suggest that acetazolamide as a carbonic anhydrase inhibitor reduces cerebrospinal fluid production and repairs the dura mater, possibly preventing fistula recurrence [17].

In our study, each 1 cc less drainage of CSF continued to increase the development of immediate-onset PDPH statistically significantly. Less CSF drainage may not increase the CSF production rate sufficiently and may lead to an earlier onset of PDPH symptoms [3,18]. Some studies show that the collection of lower volumes (especially below 17 mL) may be attributable to higher rates of follow-up headaches and therapeutic blood patches [9]. High volume loss may facilitate early dural closure by stimulating compensatory mechanisms. Similar to our results, it has been mentioned in the literature that the compensatory venodilation mechanism may not be activated when less CSF is drained because there is no significant decrease in CSF pressure [3]. In addition, underdrainage may not sufficiently increase the rate of CSF production and may lead to immediate onset of PDPH symptoms.

PDPH developed in 44.8% of our patients, and the rates in previous studies ranged from 10% to 60%, depending on the patient and procedure-related variables [19,20]. Needle diameter may be one of the most important factors underlying the higher frequency of PDPH in our study. Studies show that the frequency of PDPH development is 40%–70% with a 16–18 G needle, which is similar to our PDPH rate [18,21].

In the present study, we showed an association between high CSF chloride concentration and the development of PDPH. It is a widely accepted assumption that biochemical and microbiological changes affect the viscosity and the flow of CSF [22,23]. The effect of CSF chloride concentrations in our study may have been due to the mechanisms related to CSF production from the choroid plexus. Even in the absence of leakage, PDPH may be observed as a result of volume reduction due to electrochemical disruption of CSF production. Therefore, this result regarding chloride should be examined in further physiology studies to increase the level of evidence. Low serum ferritin levels were a risk factor for PDPH in our study. There are studies suggesting a relationship between iron deficiency anemia, low serum ferritin levels, and the incidence of migraine [24,25]. Low ferritin has also been associated with chronic daily headaches [26]. Our findings also indicate that low ferritin levels may play a role in pain processing. Primary headaches such as migraine and tension-type headaches are thought to be associated with low ferritin and iron deficiency [25]. Iron deficiency has been linked to an increased prevalence, frequency, and severity of migraines, particularly among women [27]. Migraine has also been associated with elevated cholesterol levels, and recent studies have found it to be more associated with triglycerides, although there is only indirect evidence [28]. In our study, we aimed to investigate the relationship between PDPH and iron deficiency, hypothesizing a mechanism similar to that underlying its association with primary headache disorders. We aimed to evaluate all potential risk factors, including comorbidities, transport mechanisms, and blood parameters that may influence healing. Future studies are needed.

Consistent with the literature, female sex and age were also significant risk factors, as well as needle diameter. Each 10-year decrease in age increased the probability of developing PDPH significantly. The risk was approximately 2.5 times higher in women than in men. In the literature, it has been shown that the frequency decreases after the age of 40 years, and the incidence in the 25–40 age range is 3–5 times higher compared to in the population over 60 years of age [29–31]. In the pathophysiology, inadequate response of the cerebral vessels to CSF hypotension, low CSF leakage from the subarachnoid space due to the small amount of CSF accumulation in the extradural space, and decreased elasticity of the dura with increasing age can be mentioned [32–34].

Female sex is an independent risk factor in the development of PDPH [4,18,31]. This may be due to differences in pain perception, psychosocial factors, differences in dura elasticity, estrogen-induced increase in substance P receptor reactivity, and hormone-related changes in cerebral vascular structure reactivity [18,32]. The mean anxiety score was higher in PDPH patients compared to in patients without PDPH. In previous studies, reported anxiety levels in patients with and without PDPH were controversial [29,35]. Informing the patients about the PDPH risk factors may help reduce their anxiety levels.

The strengths of our study are that since it was a single-center prospective study, the same diameter needle was used in all LP procedures, and the LP procedures were performed by neurologists with similar levels of experience. In addition, headache characteristics were evaluated by headache experts. Since it was a prospective and single-center study, the data loss observed in retrospective studies was not observed in the present study, and all headache characteristics of the patients were recorded. Since the needle characteristics most commonly associated with PDPH were not variables in our study, other procedure-related characteristics were evaluated more thoroughly. The assessment is reliable because the researchers of the study were not involved in the LP procedure. The limitation of our study was that the diagnosis of PDPH was determined only clinically. We did not evaluate intraventricular pressure or radiologically determine the presence of definitive CSF leak/intracranial hypotension in the diagnosis of PDPH. Closing pressure was measured in very few patients. Therefore, no comment was made about it. Having different preliminary diagnoses as LP indications had no effect in the development of PDPH. However, more homogeneous groups or a larger sample size in some subgroups could have increased the statistical power of the study.

## 6. Conclusion

We showed that patient-related factors may be more critical in terms of the development of PDPH and procedure-related factors gain importance in terms of the time of occurrence of PDPH. These results may help to elucidate the pathophysiology of PDPH. Determination of the risk factors for PDPH may help to provide an earlier and more accurate diagnosis and the optimization of clinical practice.

Beyond the presence or absence of headache, the CSF cycle may decompensate with the maladaptation caused by the chronicity of prior headaches. PDPH occurring within the first 24 h seems to occur secondary to volume change and the role of compensatory mechanisms is at the forefront in immediate PDPH pathophysiology. The significant effect of CSF volume change in the evaluation of acute pain starting within the first 24 h may be an objective finding of this decompensation. The risk factor differences between the occurrence of PDPH and the time of PDPH suggest that different pathophysiological processes are involved in its occurrence and timing.

Our study provides evidence that central sensitization and conditions affecting CSF dynamics and compensation mechanisms are associated with PDPH. However, further research is required to validate this hypothesis and to explore the pathophysiological mechanisms in detail.

## Figures and Tables

**Figure:**
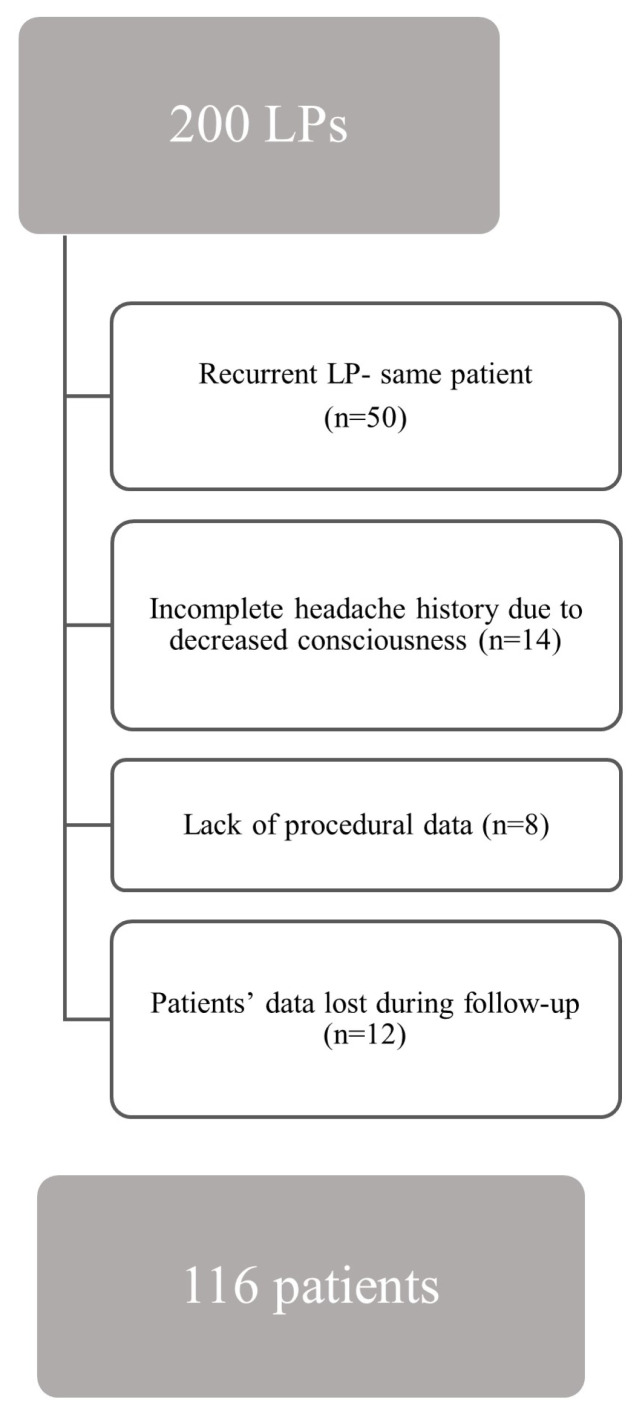
Flowchart of the study.

**Table 1:** Demographic features of the patients.

	Total (n=116)	Non-PDPH (n=64)	PDPH (n=52)	p-value	Onset time p-value
**Age (years)**	40.1±13.8	43.1±14.8	36.5 ± 11.5	**0.01** †	0.14†
**Age groups**				0.11‡	0.40#
<30	30 (%25.9)	15 (%23.4)	15 (%28.8)		
30–39	34 (%29.3)	16 (%25.0)	18 (%34.6)		
40–49	24 (%20.7)	12 (%18.8)	12 (%23.1)		
≥50	28 (%24.1)	21 (%32.8)	7 (%13.5)		
**Sex**				**0.01** ‡	0.37¶
Female	58 (%50.0)	25 (%39.1)	33 (%63.5)		
Male	58 (%50.0)	39 (%60.9)	19 (%36.5)		
**Preliminary diagnosis**					
Demyelinating disease	46 (%39.7)	24 (%37.5)	22 (%42.3)	0.74¶	>0.1¶
Idiopathic intracranial hypertension (IIH)	20 (%17.2)	9 (%14.1)	11 (%21.2)	0.45¶	**0.04** ¥
Encephalitis	10 (%8.6)	8 (%12.5)	2 (%3.8)	0.18¥	0.50¥
Normal pressure hydrocephalus	5 (%4.3)	4 (%6.3)	1 (%1.9)	0.38¥	>0.1¥
Intrathecal therapy	12 (%10.3)	4 (%6.3)	8 (%15.4)	0.19¶	>0.1¥
Motor neuron disease	2 (%1.7)	2 (%3.1)	0 (%0.0)	0.50¥	
Guillain-Barré syndrome	4 (%3.4)	4 (%6.3)	0 (%0.0)	0.13¥	
Optic neuritis	8 (%6.9)	5 (%7.8)	3 (%5.8)	0.73¥	0.25¥
Multiple cranial neuropathy	4 (%3.4)	2 (%3.1)	2 (%3.8)	>0.1¥	0.50¥
Secondary headache	1 (%0.9)	1 (%1.6)	0 (%0.0)	>0.1¥	
Spastic paraparesis	5 (%4.3)	2 (%3.1)	3 (%5.8)	0.66¥	0.57¥
**Weight (kg)**	74.7 ± 14.8	76.2 ± 15.3	72.8 ± 14.0	0.22†	
**Height (m)**	1.69 ± 0.077	1.69 ± 0.069	1.68 ± 0.086	0.98†	
**Body mass index (BMI) (kg/m** **^2^** **)**	26.3 ± 4.9	26.8 ± 5.1	25.6 ± 4.7	0.21†	0.86†

† Student’s t test,

‡ Pearson’s χ^2^ test,

¶ Continuity-corrected χ^2^ test,

¥ Fisher’s exact test,

§ Mann–Whitney U test,

# Fisher–Freeman–Halton test.

PDPH: Postdural puncture headache.

**Table 2:** Comorbidities and medications of the patients.

	Total (n=116)	Non-PDPH (n=64)	PDPH (n=52)	p-value	Onset time p-value
**Procedural anxiety**		3 (2–4)	4 (3–5)	0.05§	0.92§
**Comorbidities**					
None	66 (%56.9)	35 (%54.7)	31 (%59.6)	0.73¶	
Diabetes mellitus	10 (%8.6)	6 (%9.4)	4 (%7.7)	>0.1¥	
Hypertension	7 (%6.0)	6 (%9.4)	1 (%1.9)	0.13¥	
Rheumatologic disease	4 (%3.4)	2 (%3.1)	2 (%3.8)	>0.1¥	
Coroner artery disease	4 (%3.4)	3 (%4.7)	1 (%1.9)	0.63¥	
Thyroid disease	5 (%4.3)	2 (%3.1)	3 (%5.8)	0.66¥	
Stroke	6 (%5.2)	5 (%7.8)	1 (%1.9)	0.22¥	
Cancer	6 (%5.2)	5 (%7.8)	1 (%1.9)	0.22¥	
Spinal muscular atrophy	14 (%12.1)	6 (%9.4)	8 (%15.4)	0.48¶	
Other	13 (%11.2)	8 (%12.5)	5 (%9.6)	0.85¶	
**Medications**					
None	74 (%63.8)	39 (%60.9)	35 (%67.3)	0.61¶	
Antidiabetics	8 (%6.9)	5 (%7.8)	3 (%5.8)	0.73¥	
Antihypertensives	9 (%7.8)	7 (%10.9)	2 (%3.8)	0.18¥	
Antilipidemics	2 (%1.7)	1 (%1.6)	1 (%1.9)	>0.1¥	
Antiaggregants	15 (%12.9)	11 (%17.2)	4 (%7.7)	0.22¶	
Levothyroxine	5 (%4.3)	2 (%3.1)	3 (%5.8)	0.66¥	
Antidepressants	8 (%6.9)	3 (%4.7)	5 (%9.6)	0.46¥	
Topiramate	4 (%3.4)	0 (%0.0)	4 (%7.7)	**0.04** ¥	0.3¥
Acetezolamide	7 (%6.0)	2 (%3.1)	5 (%9.6)	0.24¥	**0.01** ¥
Other	21 (%18.1)	11 (%17.2)	10 (%19.2)	0.97¶	

† Student’s t test,

‡ Pearson’s χ^2^ test,

¶ Continuity-corrected χ^2^ test,

¥ Fisher’s exact test,

§ Mann Whitney U test,

# Fisher-Freeman-Halton test

PDPH: Postdural puncture headache.

**Table 3:** The characteristics of previous headaches.

	Total (n=116)	Non-PDPH (n=64)	PDPH (n=52)	p-value	Onset time p-value
**History of previous headache**				0.17†	> 0.1†
No	47 (%40.5)	30 (%46.9)	17 (%32.7)		
Yes	69 (%59.5)	34 (%53.1)	35 (%67.3)		
**Type of headache**					
Migraine	36 (%52.2)	16 (%47.1)	20 (%57.1)	0.55†	>0.1†
Tension type	12 (%17.4)	5 (%14.7)	7 (%20.0)	0.79†	0.2‡
Idiopathic intracranial hypertension	14 (%20.3)	6 (%17.6)	8 (%22.9)	0.81†	0.05‡
**Headache attributes**				> 0.1†	0.24†
**Location**				> 0.1†	>0.1†
**Accompanying symptoms**					
None	22 (%31.9)	14 (%41.2)	8 (%22.9)	0.17†	0.10‡
Nausea and vomiting	29 (%42.0)	12 (%35.3)	17 (%48.6)	0.38†	0.13†
Phonophobia	30 (%43.5)	11 (%32.4)	19 (%54.3)	0.11†	0.11†
Photophobia	31 (%44.9)	11 (%32.4)	20 (%57.1)	0.07†	0.52†
Osmophobia	14 (%20.3)	3 (%8.8)	11 (%31.4)	**0.04** †	0.72‡
Blurred vision	13 (%18.8)	5 (%14.7)	8 (%22.9)	0.58†	0.25‡
**Headache frequency**				**0.04** ¶	**0.02** †
> Once a week	41 (%59.4)	19 (%55.9)	22 (%62.9)		
1–2 per month	20 (%29.0)	14 (%41.2)	6 (%17.1)		
3–4 per year	6 (%8.7)	1 (%2.9)	5 (%14.3)		
1–2 per year	2 (%2.9)	0 (%0.0)	2 (%5.7)		
**Duration of the headache attack**				**0.04** §	0.21†
<2 hours	26 (%37.7)	16 (%47.1)	10 (%28.6)		
3 h to 3 days	33 (%47.8)	11 (%32.3)	22 (%62.8)		
>3 days	10 (%14.5)	7 (%20.6)	3 (%8.6)		
**Visual analogue scale score (VAS) (1–10)**	6.0 (5.0–8.0)	6.5 (5.0–8.0)	6.0 (5.0–8.0)	0.79¥	0.07¶
**Analgesic intake**				0.54¶	0.40†
**Duration of the prior headache disorder**				**0.03** ¶	0.59‡
<1 year	5 (%7.2)	5 (%14.7)	0 (%0.0)		
1–5 years	29 (%42.1)	11 (%32.4)	18 (%51.4)		
<6 years	35 (%50.7)	18 (%52.9)	17 (%48.6)		

† Continuity-corrected χ^2^ test,

‡ Fisher’s exact test,

¶ Fisher–Freeman–Halton test,

§ Pearson’s χ^2^ test,

¥ Mann–Whitney U test.

PDPH: Postdural puncture headache.

**Table 4:** The most determining risk factors.

PDPH	Odds ratio	%95 confidence interval		p-value
		Lower	Upper	
**Age** *	1.45	1.06	1.99	0.02
**Female**	2.49	1.09	5.65	0.03
**Migraine accompanied by osmophobia**	3.42	1.01	11.52	0.05
**Duration of headache**	1.61	1.02	2.53	0.04
**Immediate-onset PDPH**				
**Pre-LP diagnosis of IIH compared to those without pre-LP headache**	10.22	1.15	91.10	0.04
**The amount of CSF collected (1 cc)** **	1.15	1.01	1.31	0.03

* The effect of each 10-year decrease in age.

** Lower volume of cerebrospinal fluid collected (every 1 cc)

PDPH: Postdural puncture headache, LP: Lumbar puncture, IIH:Idiopathic intracranial hypertension, CSF: Cerebrospinal fluid

**Table 5:** Procedure-related risk factors.

	Total (n=116)	Non-PDPH (n=64)	PDPH (n=52)	p-value	Onset time p-value
**Localization**				0.13‡	
**Position**				0.25¶	
**Number of attempts**	2 (1–3)	2 (1–2)	1 (1–2)	**0.01** †	0.29†
**Traumatization**	47 (%40.5)	24 (%37.5)	23 (%44.2)	0.59¥	0.66¥
**Pressure (cm-H20)**	15.0 (12.0–23.0)	18.2 (13.2–23.0)	15.0 (11.2–24.0)	0.47†	>0.1†
**Collected CSF (cc)**	14.5 (10.0–18.7)	15.0 (10.0–20.0)	14.0 (10.0–17.0)	0.33†	

† Mann–Whitney U test,

‡ Pearson’s χ^2^ test,

¶ Fisher’s exact test,

¥ Continuity-corrected χ^2^ test.

PDPH: Postdural puncture headache.
